# Health and Fitness Apps for Hands-Free Voice-Activated Assistants: Content Analysis

**DOI:** 10.2196/mhealth.9705

**Published:** 2018-09-24

**Authors:** Arlene E Chung, Ashley C Griffin, Dasha Selezneva, David Gotz

**Affiliations:** 1 Division of General Medicine & Clinical Epidemiology Department of Medicine University of North Carolina School of Medicine Chapel Hill, NC United States; 2 Division of General Pediatrics and Adolescent Medicine Department of Pediatrics University of North Carolina School of Medicine Chapel Hill, NC United States; 3 Program on Health and Clinical Informatics University of North Carolina School of Medicine Chapel Hill, NC United States; 4 Carolina Health Informatics Program University of North Carolina at Chapel Hill Chapel Hill, NC United States; 5 School of Information and Library Science University of North Carolina at Chapel Hill Chapel Hill, NC United States

**Keywords:** voice-activated assistant, intelligent personal assistant, virtual personal assistant, Amazon Alexa, Google Assistant, artificial intelligence, voice-activated technology, voice assistant

## Abstract

**Background:**

Hands-free voice-activated assistants and their associated devices have recently gained popularity with the release of commercial products, including Amazon Alexa and Google Assistant. Voice-activated assistants have many potential use cases in healthcare including education, health tracking and monitoring, and assistance with locating health providers. However, little is known about the types of health and fitness apps available for voice-activated assistants as it is an emerging market.

**Objective:**

This review aimed to examine the characteristics of health and fitness apps for commercially available, hands-free voice-activated assistants, including Amazon Alexa and Google Assistant.

**Methods:**

Amazon Alexa Skills Store and Google Assistant app were searched to find voice-activated assistant apps designated by vendors as health and fitness apps. Information was extracted for each app including name, description, vendor, vendor rating, user reviews and ratings, cost, developer and security policies, and the ability to pair with a smartphone app and website and device. Using a codebook, two reviewers independently coded each app using the vendor’s descriptions and the app name into one or more health and fitness, intended age group, and target audience categories. A third reviewer adjudicated coding disagreements until consensus was reached. Descriptive statistics were used to summarize app characteristics.

**Results:**

Overall, 309 apps were reviewed; health education apps (87) were the most commonly occurring, followed by fitness and training (72), nutrition (33), brain training and games (31), and health monitoring (25). Diet and calorie tracking apps were infrequent. Apps were mostly targeted towards adults and general audiences with few specifically geared towards patients, caregivers, or medical professionals. Most apps were free to enable or use and 18.1% (56/309) could be paired with a smartphone app and website and device; 30.7% (95/309) of vendors provided privacy policies; and 22.3% (69/309) provided terms of use. The majority (36/42, 85.7%) of Amazon Alexa apps were rated by the vendor as mature or guidance suggested, which were geared towards adults only. When there was a user rating available, apps had a wide range of ratings from 1 to 5 stars with a mean of 2.97. Google Assistant apps did not have user reviews available, whereas most of Amazon Alexa apps had at least 1-9 reviews available.

**Conclusions:**

The emerging market of health and fitness apps for voice-activated assistants is still nascent and mainly focused on health education and fitness. Voice-activated assistant apps had a wide range of content areas but many published in the health and fitness categories did not actually have a clear health or fitness focus. This may, in part, be due to Amazon and Google policies, which place restrictions on the delivery of care or direct recording of health data. As in the mobile app market, the content and functionalities may evolve to meet growing demands for self-monitoring and disease management.

## Introduction

Hands-free voice-activated assistants (VAAs) have recently gained popularity with the release of commercial products, including Amazon Alexa and Google Assistant and their associated speaker devices. VAAs are also referred to as “intelligent personal assistants,” “voice assistants,” or “virtual personal assistants” [1]. A voice-activated assistant is a software agent that can perform tasks or services for an individual and uses voice activation for interaction through a smart speaker device. Apple’s Siri is an example of a VAA, but requires the user to press a button before one can use voice for interaction. However, hands-free VAAs allow the user to command the speaker device without having to touch the device and by using only their voice. Historically, VAA technologies have been able to perform a range of rudimentary tasks delegated by the user with the primary functions being to organize and manage information [2], such as provide facts or play music. With advancements in machine learning, artificial intelligence, and natural language processing, VAAs can now handle more complex interactions, such as commanding smart home devices and placing orders for merchandize [3-8].

Released in the United States in November 2014, the Amazon Echo was the first commercially available hands-free device controlled by voice interaction. Alexa is the cloud-based, personal voice assistant integrated into Amazon’s VAA devices, which include the Echo, Dot, Tap, Look, Spot, and Show. Amazon has a Skills Store that houses “skills,” which are apps that “…add new capabilities that create a more personalized experience with your Alexa-enabled devices…” [9]. The Skills Store is similar to the iOS and Android app stores and allows a variety of skills to be “enabled” for use on Amazon Alexa devices. Although many of them are free to “enable,” some have associated fees or require accounts to use the skills. Google Assistant is the voice assistant powering Google Home, which was released in November 2016 in the United States. In addition to Google Home, Google Home Mini and Google Home Max devices were released in late 2017. Similar to the Amazon Alexa-powered devices, Google Assistant has in-house and third-party apps called “actions.” As with Amazon, some apps require that the user link a mobile phone app account to their Google account before using the service with the Google Assistant. Most Google Assistant apps are already enabled by default. Both Amazon Alexa and Google Assistant have platforms for developers to create apps.

There are a number of potential health-related use cases for VAAs because they could be used in a variety of settings (eg, patients’ homes or in clinics and hospitals) for many different functions, such as home monitoring of symptoms or health education. Evidence suggests that VAAs could increase accessibility to information for those with physical, sensory, and cognitive impairments and facilitate self-management or education [6,8,10-13]. Prior research concerning use cases for VAAs have featured health tracking and monitoring, assistance with locating health providers, and collecting data to aid in decision making [3-5], but VAAs studied were not commercially available products. At this writing, there are only a few studies that have utilized a commercially available VAA for a health use case. These studies have used Amazon Alexa to assess deaf speech [14], provide task support for individuals with cognitive disabilities [15], and receive voice input from patients to determine “unexpected changes in mood” [5]. Additionally, a research study using Amazon Alexa was recently launched to increase physical activity among overweight or obese cancer survivors [16]. There is also limited research on the attitudes of patients or health care providers regarding the use of VAAs for health or fitness. Two studies based on customer reviews indicated that users are interested in potentially utilizing these devices for self-management, as a memory aid, or overcoming accessibility issues [12,13].

Although there is rich literature regarding the characteristics and use of health and fitness mobile phone apps [17-25], no prior studies have described the characteristics of health and fitness apps for commercially available hands-free VAAs. Previous VAA studies have focused only on research-grade VAAs that were not commercially available, were not hands-free, and were primarily focused on usability and design of the devices rather than the apps that could be used with VAAs [1,2,6,7,10,26,27]. Thus, this study is the first to examine the features and characteristics of hands-free commercially available VAA apps for health and fitness based on information available from app marketplaces.

## Methods

### Selection Criteria and Methodology

VAA apps are uniquely different from mobile phone apps, in that the full scope of the types of interactions are not clearly delineated by interacting with the voice interface because it does not have the same transparency as interacting with a physical user interface (eg, the screen of a mobile phone). VAA vendors generally provide only a few examples of invocation commands, and there is no menu of features or functionalities such that the user or evaluator could understand the full spectrum of the types of commands one could ask the VAA app or what types of information could be provided by the app. Thus, these aspects of VAAs and voice-based interfaces do not allow for the direct application of the traditional review methods used for mobile health apps. Because there were no review methodologies specific to VAA apps, we used existing methods for evaluating the content of mobile health apps for guidance [17-21,28]. This study specifically focused on conducting a descriptive content analysis based on the information provided by vendors to determine the types of apps released in the health and fitness categories for commercially available VAAs because this is the information consumers use to select apps from VAA app marketplaces.

As of the review date April 19, 2017, Amazon and Google were the only companies with commercially available hands-free VAAs (Amazon Alexa and Google Assistant). Thus, the Amazon Alexa Skills Store website and mobile phone app (iOS and Android) and Google Assistant mobile phone app (iOS and Android) were searched to determine the availability of eligible voice apps. The full list of categorized voice apps for the Google Assistant was only available through the Google Assistant mobile phone app (iOS and Android). There were 23 types of VAA app categories listed on the Alexa Skills Store and 17 categories listed on the Google Assistant mobile phone app.

**Figure 1:**
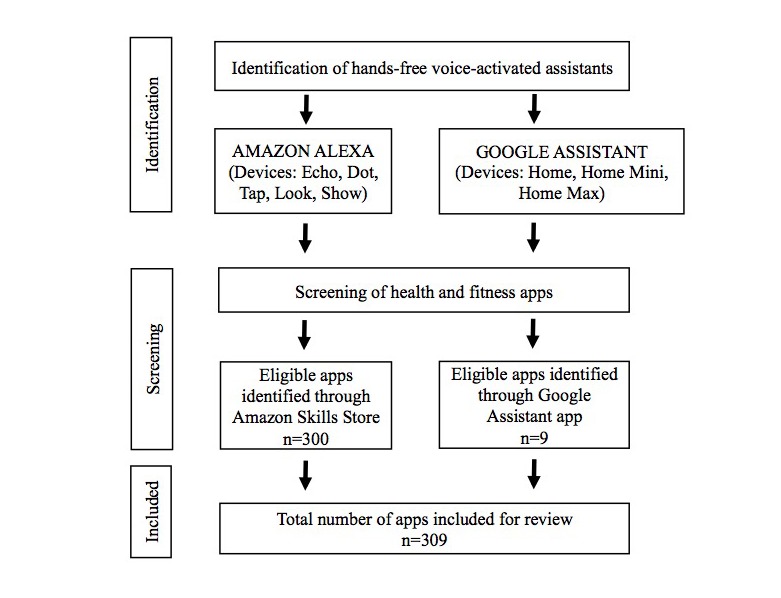
Health app identification, screening, and review assessment flowchart.

Inclusion criteria included any VAA apps that were categorized by vendors in the “health and fitness” categories. Both Amazon Alexa and Google Assistant have a “health and fitness” category for apps, and apps could be cross-listed in multiple categories; for example, an Amazon Alexa nutrition app could be listed by the app vendor in both the health and fitness category and the food and drink category. For the apps meeting the inclusion criteria, information provided by vendors was extracted into an evaluation form (Textbox 1). The Amazon Alexa skill release date was retrieved from a third-party website [9,29] but was manually verified using the dates available on the Amazon Skills Store. The Google Assistant app release dates were not publicly available except for a press release, which listed all the apps released as of April 19, 2017. Thus, this was the date selected for data extraction. Figure 1 presents a flowchart for identification, screening, and review of apps.

### Analysis

A codebook of categories for the type of health and fitness app, intended age group, and the target audience was created to evaluate each app (Table 1). Codebook development was guided by definitions from mobile phone app reviews of app content [18,22] because there were no VAA app reviews to use for guidance. Intended age was used to classify the age group that the app was designed for, and the target audience was used to classify the population most likely to use the app or be the end user [23]; for example, a baby monitoring app would be coded with an intended age category of children, and the target audience would be parents or families. If a vendor provided a rating of “guidance suggested” or “mature,” these were coded as for adults because Amazon states these types of apps contain nudity, violence, references to substance use, profanity, or sexuality and that these apps are for adults only [23]. Google apps did not provide any vendor ratings.

A single app could be coded into multiple health and fitness, intended age, and target audience categories. The health and fitness categories for baby naming, beauty tips, baby monitoring and tracking, dog monitoring and tracking, brain training and games, and time and task management were added during the iterative coding process.

**Extracted vendor information.**

General information

- App name
- Release date, if available
- Cost to enable or link app to voice-activated assistant device
- Vendor rating (mature audience or guidance suggested), if available

Vendor information

- Name of the developer
- Has a developer policy
- Has a privacy policy

User ratings

- Number of user reviews, if available
- User rating from 1-5 stars

Features

- Ability to pair with a mobile phone app, website, or device
- Description of the app
- Example voice interaction or invocation word(s)

**Table 1:** Definitions of health and fitness, target audience, and intended age group categories.

Name of Category		Definition
**Health and** **fitness category**		
	Air quality and monitoring	Inform users about air condition, air quality, or pollutants
	Baby naming	Provide suggestions for baby names
	Baby monitoring and tracking	Track feeding, diaper changes, naps, or other baby and toddler events
	Beauty tips	Provide tips for beauty topics, such as hair, skin, and foot care
	Brain training and games	Contain games or tests for memory or mental awareness
	Care giving	Provide information for caregivers
	Diet and caloric intake	Track calories, food, or beverage intake
	Dog monitoring and tracking	Track feeding, walks, sleep, or other dog events like stools
	Fitness and training	Provide workout plans, improve physical fitness, or assist with training
	Global positioning system or geographic information system	Track location or assist with navigation to locations
	Health education	Provide information or tips about health and wellness topics
	Health location	Locate medical resources, fitness centers, or wait times at medical facilities
	Health monitoring	Review vital signs or labs (blood pressure, heart rate, height, weight, body mass index, blood sugars, etc) and track medications or symptoms
	Immunization tracking	Track immunizations
	Meditation	Provide practices or techniques to promote relaxation, build internal energy, or mindfulness
	Mental health	Provide information on mental health issues, such as depression or anxiety
	Motivational	Provide guidance on the desire to perform a specific action or behavior
	Nutrition	Provide advice or information on dietary topics, meal or snack planning, etc
	Pain management	Inform users about pain management relief strategies
	Pregnancy tracking	Provide guidance for users who are planning or expecting to have a baby
	Stress management	Inform users about ways to manage stress
	Sleep	Inform users on ways to improve sleep or to track sleep
	Smoking cessation	Provide information about smoking cessation strategies
	Time or task management	Provide strategies for tracking or managing time and tasks
	Other	Does not fall into any of the above categories
**Target audience (end user)**		
	Family	Family relations (parents, child, extended family)
	Medical professional	Person who provides medical care (doctor, nurse, etc)
	Parent	Person with one or more children
	Patient	Person seeking medical care or has a medical condition
	Pregnant women	Woman expecting a baby
	Women	For women
	Men	For men
	Pet owner	Person with one or more pets
	Student	Person who attends an educational institution
	General or not specified	Does not specify an intended user
	Other	Does not fall into any of the above categories
**Intended age group**		
	Adult^a^	≥18 years
	Children	Newborn to <18 years
	Older adults	≥65 years
	Not specified	Does not specify a target age

^a^The vendor rating guidelines from Amazon include the following definitions: 1) guidance suggested: may have nudity or suggestive content or require supervision due to account linking, location detection, etc and 2) mature: content is for adults only. Any app that had a vendor rating of guidance suggested or mature was coded as for adults. Additionally, apps that specifically mention adults in the app description were coded for adults.

Two reviewers (DS and ACG) independently coded each app based upon information provided by vendors into one or more health and fitness categories, intended age groups, and target audience groups. Any discordant codes were reconciled through discussion with a third reviewer (AEC) until consensus was reached. Descriptive statistics were used to summarize the number of apps in each of the health and fitness, intended age, and target audience categories. Additionally, the interrater agreement between primary coders was calculated.

## Results

There were 309 apps in total that met inclusion criteria for being listed as “health and fitness” apps by vendors, as seen in Figure 1. We identified 300 apps from the Amazon Skills Store website and Amazon Alexa mobile phone app, and 9 apps were identified from the Google Assistant mobile phone app. Of the 9, 5 Google apps were also available in the Amazon Alexa Skills Store. The percent agreement for coding between raters was 91%.

Apps reviewed had a release date between November 6, 2015 and April 19, 2017. The Google Assistant app store did not have any user reviews available. With respect to Amazon Alexa apps, 174 apps had user reviews with a total of 1862 reviews ranging from 1 to 447 reviews per app (Table 2). On average, health and fitness apps were rated 2.97 out of 5 stars. All apps were free to enable, though some required an associated account, which may or may not charge a subscription fee. Some of the apps could be paired with a mobile phone app, website, or a device (56/309, 18.1%).

**Table 2:** Health and fitness app characteristics^a^.

App characteristics		Google (n=9)		Amazon (n=300)		Total (N=309)	
**Vendor rating^b^, n (%)**							
	Guidance suggested		0 (0)		33 (11.0)		33 (10.7)
	Mature		0 (0)		3 (1.0)		3 (1.0)
	Not available		9 (100.0)		264 (88.0)		273 (88.3)
**User rating (1-5 stars), n (%)**							
	1-1.9		0 (0)		41 (13.7)		41 (13.2)
	2-2.9		2 (22.2)		41 (13.7)		43 (13.9)
	3-3.9		6 (66.7)		30 (10.0)		36 (11.7)
	4-5		0 (0)		62 (20.6)		62 (20.1)
	Not available		1 (11.1)		126 (42.0)		127 (41.1)
**User reviews^b^, n (%)**							
	1-9		0 (0)		152 (50.7)		152 (49.2)
	10-99		0 (0)		18 (6.0)		18 (5.8)
	≥100		0 (0)		4 (1.3)		4 (1.3)
	Not available		9 (100.0)		126 (42.0)		135 (43.7)
Cost: free to enable		9 (100.0)		300 (100.0)		309 (100.0)	
Has a developer policy		5 (55.6)		64 (21.3)		69 (22.3)	
Has a privacy policy		9 (100.0)		86 (28.7)		95 (30.7)	
Ability to pair with a mobile phone app, website, or device		3 (33.3)		53 (17.7)		56 (18.1)	

^a^There were 309 apps evaluated. Apps could be included in multiple categories and were not mutually exclusive.

^b^Google Assistant does not provide vendor ratings or user reviews. The vendor rating guidelines from Amazon include: 1) guidance suggested: may have nudity or suggestive content or require supervision due to account linking, location detection, etc, and 2) mature: content is for adults only.

**Table 3:** Number of apps by health category, target audience, and age group (N=309). Apps could be included in multiple categories and were not mutually exclusive.

Categories		Google	Amazon	Total
**Health category**				
	Health education	3	84	87
	Fitness and training	3	69	72
	Nutrition	0	33	33
	Brain training and games	1	30	31
	Health monitoring	0	25	25
	Motivational	0	22	22
	Meditation	0	16	16
	Other	0	15	15
	Health location	0	15	15
	Stress management	0	12	12
	Global positioning system or geographic information system	0	9	9
	Diet and caloric tracking	0	9	9
	Sleep	0	8	8
	Mental health	0	7	7
	Air quality monitoring	1	6	7
	Baby monitoring and tracking	0	5	5
	Smoking cessation	1	5	6
	Baby naming	2	3	5
	Care giving	0	4	4
	Time and task management	1	2	3
	Pregnancy tracking	0	3	3
	Dog Monitoring and tracking	0	2	2
	Beauty tips	0	2	2
	Immunization tracking	0	1	1
**Target audience**				
	General or not specified	5	261	266
	Patients	1	32	33
	Parents	2	14	16
	Family	1	13	14
	Medical professionals	0	6	6
	Pregnant women	0	4	4
	Pet owners	1	2	3
	Women	0	2	2
	Other	0	1	1
	Men	0	0	0
**Intended age**				
	Not specified	8	249	257
	Adults	0	42	42
	Children	1	9	10
	Older adults	0	1	1

Collectively, 10.7% (33/309) of apps were rated by the vendors as “guidance suggested” and 1.0% (3/309) were rated as “mature.” Furthermore, 30.7% (95/309) of apps had a privacy policy, and 22.3% (69/309) had a developer’s policy for terms of use.

The most frequently occurring types of health and fitness VAA apps were health education (87), fitness and training (72), nutrition (33), brain training and games (31), health monitoring (25), motivational (22), and meditation (16), as seen in Table 3. The most common target audience (population that would use the app) was general or not specified (266), followed by patients (33), parents (16), and families (14). In terms of the intended age group, 257 apps did not have age specified, and there were 42 apps focused on adults, 10 focused on children, and 1 focused on older adults (Table 3). The majority of Amazon’s apps coded for adults were rated by the vendor as mature or guidance suggested (36/42, 85.7%).

## Discussion

### Principal Findings

In this study, which is the first to report an analysis of VAA apps for health and fitness, we found that although the marketplace appeared to have many apps in these topical areas, there were mainly apps that focused on either health education or fitness and training and many did not seem to actually have a clear health or fitness focus at all (eg, baby naming). These apps were mostly targeted toward adults and general audiences with only a few apps specifically geared toward older adults or those with disabilities. However, these are the populations that may potentially benefit the most from VAA technologies [8,10,15]. Strikingly, very few apps were categorized as care giving or targeted specifically toward patients, caregivers, or medical professionals, yet these populations could be supported by VAAs; for example, the use of non-commercial VAAs in assisted living facilities has been associated with higher quality of living and improved recovery from illness [6,10]. Additionally, VAA apps that focus on social interaction and support, communication, care coordination, reminders, remote monitoring, locating providers, and scheduling appointments and transportation could be potentially impactful for both patients and their caregivers, but there are currently limited VAA apps available for these purposes. The main potential barriers to advancing the use of VAA apps for health are the restrictions and limitations for publishing VAA apps in the marketplace, security and privacy issues, and the credibility of these apps.

### Health Monitoring

Our analysis revealed there was only a limited number of health monitoring apps (25). This may be, in part, due to the restrictions within Amazon and Google policies for publishing health-related apps. Amazon does not allow apps to be certified for release in the app store if the app “collects information relating to any person’s physical or mental health or condition, the provision of health care to a person, or payment for the same;” “does not include a disclaimer in the skill description stating that the skill is not a substitute for professional advice;” and “claims to provide life-saving assistance through the skill or in the skill name, invocation name, or skill description” [30]. Thus, associated mobile phone apps could be used to collect health data to be reviewed within the VAA app, but the VAA app does not ask the user to directly document health data, such as blood pressure readings, using the voice interface. Google advises that “health care providers, health plans, or health care clearinghouses wishing to develop an action should be aware that Google is not able to commit that the actions on Google platform meet the requirements of the Health Insurance Portability and Accountability Act (HIPAA) or other relevant legal provisions [31].” There is no specific information regarding HIPAA on the Amazon website. More clarity and guidance in interpreting policy restrictions would help developers better understand what is possible and allowed for release to the market; for example, it is unclear whether biometric data collected outside of the VAA via a wearable device or mobile phone app but not directly captured by the VAA is allowable under Amazon’s restrictions. Ultimately, HIPAA will need to be addressed if health data are collected via VAAs and integrated into electronic health records similar to other patient-generated health data from devices. These restrictions may be barriers to actualizing the potential benefits of leveraging VAAs for health care because VAAs could be used to collect health data for remote monitoring and to deliver care.

### Security and Privacy

Only a small proportion of vendors provided privacy (95/309, 30.7%) or terms of use (69/309, 22.3%) policies (Table 2). Although voice recognition technologies could potentially restrict access to a specific person, an unauthorized user could still gain access to account information. Google Assistant was the first to distinguish between various users’ voices and provides different levels of security access to the Google Home device through multiple user accounts. Amazon recently followed with a similar feature.

There are a number of potential security and privacy issues when using VAAs. However, customers may not recognize or realize the security or privacy implications of using VAA devices. Both the Amazon and Google voice-activated devices remain in a passive listening state for a specific keyword or “wake” word to activate the device to begin recording and transmitting audio; for example, Burger King revealed a vulnerability of VAA devices when their television advertisement stated the wake word for the Google Assistant (“OK, Google”) and asked a question about the Whopper [32]. Additionally, because voice is a unique identifier, users should be concerned about how VAA companies collect, store, analyze, or share this information. These privacy issues were highlighted when prosecutors issued a warrant to obtain audio recordings from a murder suspect’s Amazon Echo device [33]. Although Amazon did not provide the recordings in this case and cited first amendment protection over the information gathered and sent by the device, this highlighted the fact that Amazon VAA devices record and store data. Amazon and Google Home devices have a button that can be pressed to “mute” the device from listening, but instruction manuals do not have explicit language describing that the devices are always listening and that they are recording and storing audio. Both Amazon and Google VAA devices permit the user to delete search histories and audio recordings but discourage the user from doing so as it will limit personalization. The feature to delete recordings may also not be apparent to users, and it is also unclear how long recordings are stored for. Moreover, these devices can access other accounts, which could contain private information; for example, Google Home can be enabled to access your calendar, email account, and shopping accounts, which raises additional security and privacy issues. Therefore, it is vital to have vendor transparency about when a user’s voice data are stored and transmitted and to whom and what audio and other data are recorded and stored either on the device or in the cloud and for how long.

### Developer Platforms and Application Programming Interfaces

**Figure 2:**
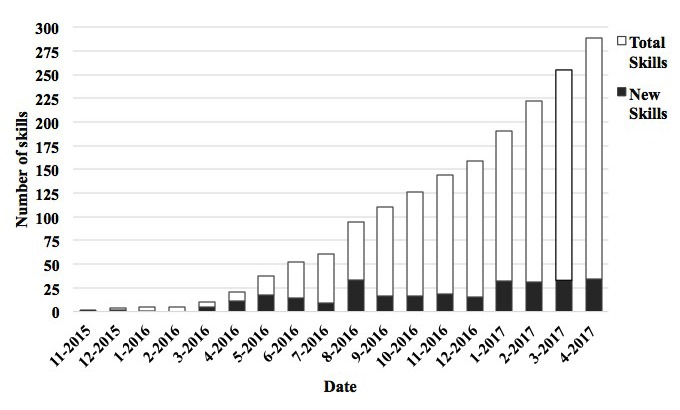
Total number of Alexa skills released over time (Alexa Skills Kit application programming interface released June 2015).

Figure 2 shows that there has been a steady increase in health and fitness apps released by Amazon since 2015 [9,29]. The addition of the Alexa Skills Kit (June 2015), a self-service API that contains a collection of tools and sample code, likely contributed to this increase because it took some time for a developer community to become established. Google Assistant released its API in December 2016. Although our search returned only 9 Google Assistant apps, there may be an uptick in release of apps over time as their developer platform and community matures. Only 18.1% (56/309) of apps examined in this study could be paired with a website, app, or device, but this is likely to expand as smart health and home devices continue to emerge (Table 2). Providing an open ecosystem with developer platforms and APIs accelerates the adoption and use of devices and the development of apps, thereby expanding the customer and market base for VAAs. Thus, future VAA devices should consider providing these self-service tools.

### Credibility

Both Amazon and Google provide only limited information about each VAA app or its features and functionalities. Similar to mobile phone apps, it is difficult to determine the credibility and value of the content of apps based solely on vendor descriptions; for example, it would be difficult for a customer to determine whether health care systems, hospitals, or providers endorsed or developed the apps unless it was explicitly noted in the app name, such as the Boston Children’s Hospital’s KidsMD app. It is also unclear whether apps were developed with user-centered design principles or evidence-based guidelines or materials, particularly for health education materials. For health-focused apps that “…meet the regulatory definition of a device but pose minimal risk to patients or consumers, the Food and Drug Administration (FDA) exercises enforcement discretions…” and does not expect manufacturers to register and list their apps with the FDA [34]. Additionally, FDA does not deem entities that distribute mobile phone apps to be medical device manufacturers. Although there are no explicit comments from FDA regarding VAAs, it is likely that the regulations related to mobile phone apps would be applied similarly to VAA apps.

Traditionally, the development of health mobile phone apps has lacked stakeholder involvement [17,18,26], which has contributed to high rates of app abandonment due to lack of usability and poor user experience. Design and usability principles should also guide VAA app development and be focused on the distinct challenges and benefits of interactive voice-based user interfaces.

To integrate VAAs into health care, the design of voice-enabled user interfaces must consider the various needs of the end user, the setting (eg, background noise and multiple users of a single device in a home, such as the patient, their caregiver, home health nurse, etc), and the unique privacy and security issues that come with using these devices that are always listening in the background. With advancements in speech recognition and algorithms that enhance accuracy, VAA apps can become more sophisticated to recognize and respond to a diverse realm of users [3].

The translation of voice to text through speech recognition provides an opportunity to track and understand patient interactions and behaviors, though the integration and interoperability of the data, particularly with the electronic health record and patient portal, have not yet come to fruition but have much promise. Within health care, VAAs must take into account the context of the person to offer timely, appropriate, and valuable feedback, including the ability to understand speech, provide meaningful feedback, and generate accurate results. VAAs are also limited in their ability to complete complex tasks because users must rely on working memory instead of being able to visually browse through a Web, tablet, or mobile phone interface for cues and assistance [35]. Thus, a combination of user interactions with both VAA and mobile phone or tablet could help end users navigate more complex tasks.

As we learn more about how people use and interact with VAAs and the shortcomings of VAAs in terms of unmet needs or expectations, app designers, developers, and researchers can start to customize user experiences that align more closely to user needs and enhance usability. An exploratory ethnographic analysis of user reviews from Amazon Echo and Dot revealed a number of concepts around user experience, such as health care-related workarounds, quality of life improvement and physical disability, companionship, and benefits to health care [12]. A deeper understanding of the way patients potentially utilize VAAs for health and fitness could also help provide potential use cases for future app development and refinement of app functionality. The aforementioned challenges are important opportunities for future research.

### Limitations

There are a few limitations to our study. First, the authors only examined apps published in the “health and fitness” category, though there may potentially be other health-related apps that exist in the “smart home,” “food and drink,” or other categories in the Amazon Skills Store and Google Assistant websites or apps. Apps are released, modified, updated, and discontinued on a regular basis. As a result, there may be apps that were reviewed in this study that are no longer available, and there may have been modifications in app descriptions since data extraction. Authors also relied solely on information published by vendors on the Amazon Skills Store and Google Assistant websites and apps. Thus, it is possible that the features listed may not be present in the actual app, which is not a unique issue to our study but app stores in general. Moreover, user reviews and star ratings may change over time as additional users make submissions. As with other customer reviews and ratings, these VAA reviews and ratings may not be representative of the quality of the apps because they may not reflect the actual content or usability of these VAA apps. Despite these limitations, our study is the first to review health and fitness apps for VAAs and contributes toward an understanding of the characteristics of health and fitness apps available for commercially available, hands-free VAA.

### Implications

There are a number of key implications of this research. Because the VAA app marketplace is still evolving, a cursory look through the names of available apps may suggest that there is, in fact, a growing number of VAA apps focused on health and fitness. However, this impression is misleading, and our study findings provide a clearer picture of the number and scope of VAA apps available, which are predominantly focused on fitness, training, and health education. Understanding what is available in the marketplace also helps to illuminate where there may be a health or fitness use case or need but no apps currently available.

Additionally, we found that most apps are focused on general audiences than on specific health use cases. In general, the VAA app market does not contain as much health-focused content in comparison to the mobile health app market, where chronic disease, monitoring, and self-management apps have proliferated. In particular, VAAs offer advantages when compared with the physical user interfaces of computers and mobile devices (tablets and mobile phones) because voice is used for interactions with the app. This could improve accessibility for those with limited sight, physical limitations, limited literacy, and limited computer proficiency. VAA apps could also be used as a vehicle to deliver clinical and behavioral interventions, as a data collection tool for research, and to deliver health care, but these are also currently lacking in the marketplace.

To evolve the current market, privacy, security, and HIPAA compliance need to be addressed along with lessening the stringent requirements from publishers such that health care and direct health monitoring could potentially be enabled or delivered via hands-free VAAs. It is also likely that support for integration with apps that run on other platforms (phones, tablets, Web, medical devices, smart home devices, etc.) will be important to overcome some of the limitations of VAA technologies highlighted in this paper, to enhance the user experience, and to leverage the opportunities that stem from voice-based user interfaces. Additionally, because there is usually a proliferation of apps when there are APIs and developer platforms available, their availability will also be critical to encourage innovation and a strong user base and to enable researchers to develop VAA apps for interventions and for facilitating data collection.

### Conclusions

The emerging market of health and fitness apps for hands-free VAAs is still nascent and mainly focused in the areas of health education and fitness. As with other health technologies, the usability and credibility of health apps are critical to ensuring adoption and long-term use. Further work is necessary to evaluate the usefulness, usability, user experience, quality, privacy, and security implications of VAA apps. Future research should also consider the development of an evaluation method for VAA apps given the unique nature of the voice interface, such that the content of the apps can be assessed for quality and usability. It will also be imperative to understand workflow barriers and facilitators required to optimally integrate VAAs into clinical care contexts and within patients’ homes and lives and to determine the acceptability and feasibility of deploying VAAs for health care use cases.

## Abbreviations

API: application programming interface
FDA: Food and Drug Administration
HIPAA: Health Insurance Portability and Accountability Act
VAA: voice-activated assistant
